# Analysis of Epitopes on Dengue Virus Envelope Protein Recognized by Monoclonal Antibodies and Polyclonal Human Sera by a High Throughput Assay

**DOI:** 10.1371/journal.pntd.0001447

**Published:** 2012-01-03

**Authors:** Hong-En Lin, Wen-Yang Tsai, I-Ju Liu, Pi-Chun Li, Mei-Ying Liao, Jih-Jin Tsai, Yi-Chieh Wu, Chih-Yun Lai, Chih-Hsuan Lu, Jyh-Hsiung Huang, Gwong-Jen Chang, Han-Chung Wu, Wei-Kung Wang

**Affiliations:** 1 Institute of Microbiology, College of Medicine, National Taiwan University, Taipei, Taiwan; 2 Department of Tropical Medicine, Medical Microbiology and Pharmacology, John A. Burns School of Medicine, University of Hawaii at Manoa, Honolulu, Hawaii, United States of America; 3 Institute of Cellular and Organismic Biology, Academia Sinica, Taipei, Taiwan; 4 Tropical Medicine Center and Department of Internal Medicine, Kaohsiung Medical University Hospital, Kaohsiung, Taiwan; 5 Faculty of Medicine, College of Medicine, Kaohsiung Medical University, Kaohsiung, Taiwan; 6 Department of Health, Center for Disease Control, Taipei, Taiwan; 7 Division of Vector-Borne Diseases, Department of Health and Human Service, Center for Disease Control and Prevention, Fort Collins, Colorado, United States of America; University of Rhode Island, United States of America

## Abstract

**Background:**

The envelope (E) protein of dengue virus (DENV) is the major target of neutralizing antibodies and vaccine development. While previous studies on domain III or domain I/II alone have reported several epitopes of monoclonal antibodies (mAbs) against DENV E protein, the possibility of interdomain epitopes and the relationship between epitopes and neutralizing potency remain largely unexplored.

**Methodology/Principal Findings:**

We developed a dot blot assay by using 67 alanine mutants of predicted surface-exposed E residues as a systematic approach to identify epitopes recognized by mAbs and polyclonal sera, and confirmed our findings using a capture-ELISA assay. Of the 12 mouse mAbs tested, three recognized a novel epitope involving residues (Q211, D215, P217) at the central interface of domain II, and three recognized residues at both domain III and the lateral ridge of domain II, suggesting a more frequent presence of interdomain epitopes than previously appreciated. Compared with mAbs generated by traditional protocols, the potent neutralizing mAbs generated by a new protocol recognized multiple residues in A strand or residues in C strand/CC′ loop of DENV2 and DENV1, and multiple residues in BC loop and residues in DE loop, EF loop/F strand or G strand of DENV1. The predominant epitopes of anti-E antibodies in polyclonal sera were found to include both fusion loop and non-fusion residues in the same or adjacent monomer.

**Conclusions/Significance:**

Our analyses have implications for epitope-specific diagnostics and epitope-based dengue vaccines. This high throughput method has tremendous application for mapping both intra and interdomain epitopes recognized by human mAbs and polyclonal sera, which would further our understanding of humoral immune responses to DENV at the epitope level.

## Author Summary

Dengue virus is the leading cause of arboviral diseases worldwide. The envelope protein is the major target of neutralizing antibodies and vaccine development. While previous studies have reported several epitopes on envelope protein, the possibility of interdomain epitopes and the relationship of epitopes to neutralizing potency remain unexplored. We developed a high throughput dot blot assay by using 67 alanine mutants of surface-exposed envelope residues as a systematic approach to identify epitopes recognized by mouse monoclonal antibodies and polyclonal human sera. Our results suggested the presence of interdomain epitopes more frequent than previously appreciated. Compared with monoclonal antibodies generated by traditional protocol, the potent neutralizing monoclonal antibodies generated by a new protocol showed several unique features of their epitopes. Moreover, the predominant epitopes of antibodies against envelope protein in polyclonal sera can be identified by this assay. These findings have implications for future development of epitope-specific diagnostics and epitope-based dengue vaccine, and add to our understanding of humoral immune responses to dengue virus at the epitope level.

## Introduction

Dengue virus (DENV) belongs to the genus *Flavivirus* in the family *Flaviviridae*. The four DENV serotypes (DENV1, DENV2, DENV3, and DENV4) are the leading cause of arboviral diseases in the tropical and subtropical areas [1], [2]. It has been estimated that more than 2.5 billion people in over 100 countries are at risk of infection and more than 50 million dengue infections occur annually worldwide [1], [2]. The clinical presentations after DENV infection range from asymptomatic infection or a self-limited illness, dengue fever (DF), to severe and potentially life-threatening diseases, dengue hemorrhagic fever/dengue shock syndrome (DHF/DSS) [1], [2]. Despite the development of candidate vaccines, several being tested in clinical trials, there is no dengue vaccine currently available [3].

DENV contains a positive-sense, single-stranded RNA genome of about 10.6 kilobases in length. Flanked by the 5′ and 3′ untranslated regions, the genome has a single open reading frame encoding a polyprotein precursor, which is cleaved by cellular and viral protease into three structural proteins, the capsid, precursor membrane (prM) and envelope (E), and seven non-structural proteins [4]. DENV enters the cell via receptor-mediated endocytosis [4]–[6]. Within the endosome, a series of E protein conformational changes is triggered by low pH, leading to the fusion of viral membrane to endosomal membrane [5], [7]. Following uncoating, translation and RNA replication, assembly occurs in the rough ER membrane, and newly formed immature virions transport through the ER lumen to the secretory pathway [4], [5], [8], [9]. In the trans-Golgi, mature virions are produced after prM protein is cleaved by furin or furin-like protease, though the cleavage is often inefficient [10]–[13]. During flaviviral replication, small and slowly-sedimenting subviral particles are produced [4]. Expression of prM and E proteins together can produce recombinant virus-like particles (VLPs). The E proteins on VLPs, which had a different symmetry, were reported to be structurally and antigenically similar to those on infectious virions [14]–[16]. VLPs have been used as non-infectious serodiagnostic reagents and flavivirus vaccine candidates [17]–[20].

The E protein forms 90 “head to tail” homodimers on the surface of mature virions [4], [5], [21]. E protein participates in virus entry and is the major target of neutralizing antibodies (Abs), which correlate with the protection against infection [4], [22]. In the presence of some cross-reactive non-neutralizing anti-E Ab, DENV replicates to higher titers in human monocytes *in vitro*, a phenomenon known as antibody-dependent enhancement (ADE) [23], suggesting that E protein is also the target of enhancing Abs. X-ray crystallographic studies of the ecotodomain of E protein reveals three distinct domains [7], [24]. Domain I is located at the center. Domain II contains an internal fusion loop at the tip and is involved in membrane fusion and dimerization of E protein. Domain III is believed to participate in receptor binding [4], [5], [25].

In the genus *Flavivirus*, there are clusters of mosquito- and tick-borne viruses. The mosquito-borne viruses consist of the Japanese-encephalitis virus (JEV) serocomplex including JEV, West Nile virus (WNV), St. Louis encephalitis virus (SLEV), Murray Valley encephalitis virus (MVEV) and Kunjun virus (KUNV), the DENV serocomplex including four serotypes of DENV, and the yellow fever virus (YFV) as a single member. Among the tick-borne viruses are the tick-borne encephalitis virus (TBEV) serocomplex, including central European, Far Eastern and Powassan TBEV [4]. Anti-E Abs that recognize members from different serocomplexes, all/subset of members within a serocomplex and a single member are called flavivirus group-reactive (GR), complex/subcomplex-reactive (CR/sCR) and type-specific (TS), respectively [26]. Previous studies of mouse anti-E monoclonal antibodies (mAbs) against DENV reported that the epitopes of GR mAbs were primarily mapped to highly conserved residues at the fusion loop of domain II [27], [28]. Using a yeast surface-display library containing domain III or domain I/II alone or recombinant domain III, the epitopes of CR/sCR mAbs were mapped to domain III, whereas those of TS mAbs were also mapped to domain III [29]–[35]. However, several questions regarding the epitopes on DENV E protein remain largely unknown. A recent study revealed that potent neutralizing mAbs against JEV derived from Fabs of chimpanzees recognized residues in domain I or domain II plus those in domain III, suggesting the presence of interdomain epitope [36]. Similarly, two potent human mAbs against WNV E protein were reported to recognize residues at domain II dimer interface and domain I/II hinge region [37]. The possibility of interdomain epitopes on DENV E protein, which cannot be identified by systems using recombinant domain I/II or III alone, remains to be investigated. Additionally, the neutralizing potency of the recently reported anti-E mAbs, which were generated by a novel immunization protocol involving IFN-α/β R -/- C57BL/6 mice [33]–[35], was much higher than previously described mAbs [29], [30], [32], [38]–[42]. Whether the epitope residues recognized by these potent neutralizing mAbs differ from those recognized by less potent neutralizing mAbs remains unclear.

In this study, we generated a panel of 67 alanine-substitution mutants covering the predicted surface-exposed E residues on a DENV1 prM/E expression construct as a systematic approach to investigate the epitopes on different domains of E protein recognized by mouse mAbs and polyclonal human sera. The identified epitopes were further verified by capture-ELISA using VLPs. Moreover, analysis of the epitopes of the potent neutralizing mAbs in comparison with those of less the potent neutralizing mAbs provided new insights for future strategies of epitope-based dengue vaccine.

## Materials and Methods

### Mouse mAbs

The mouse anti-E mAbs studied included five GR mAbs (FL0232, FL0231, 4G2, DEN2-12 and DEN4-4), four DENV CR/sCR mAbs (DEN2-9, DEN3-3, DEN3-4 and DEN1-2), and three DENV1 TS mAbs (FL0251, DA6-7 and DEN1-3). FL0232, FL0231 and FL0251 were purchased from Chance Biotechnology (Taipei, Taiwan), and 4G2 from American type culture collection (Rockville, MD). Other mAbs were generated by immunization of BALB/c mice with DENV1 (DEN1-2, DA6-7 and DEN1-3), DENV2 (DEN2-12, DEN2-9), DENV3 (DEN3-3, DEN3-4) or DENV4 (DEN4-4) as described previously [38].

### Ethics Statement and Human Sera

With the approval of the Institutional Review Board of the University of Hawaii at Manoa (CHS#17568) and National Taiwan University (IRB#1003701987), convalescent sera of confirmed dengue patients from the Kaohsiung Medical University Hospital between 2002 and 2009 and from the Center for Disease Control Taiwan were included in the analysis of Abs response to DENV. Informed consent was obtained, and all samples were coded for anonymity. WHO case definitions were used to diagnosed DF and DHF [2]. Primary or secondary infection was confirmed by plaque reduction neutralizing test (PRNT) (see below). Primary infection was defined by monotypic neutralization pattern with PRNT_50_ ≥20 to only one serotype or ≥20 to multiple serotypes with PRNT_50_≥80 to only one serotype [43], [44]. Secondary infection was defined by PRNT_50_≥20 to multiple serotypes without monotypic pattern. A JEV-NS1 IgM ELISA was used to exclude recent JEV infection [44].

### Plasmid and E Mutant Constructs

The plasmid expressing prM/E proteins of DENV1 (Hawaii strain), pCB-D1, was described previously [45]. To generate E mutant constructs, site-directed mutagenesis was performed by using pCB-D1 as template and single-step PCR mutagenesis to replace each of the selected E residues with an alanine [46]. Sixty E residues predicted to be surface-exposed as well as seven domain III E residues previously reported as epitopes of several mAbs were chosen for mutagenesis [29]-[35], [47]. All mutant constructs were confirmed by sequencing the entire insert to rule out second site mutation. The sequences of all PCR primers will be provided upon request.

### Transfection and Western Blot Analysis

293T cells (1×10^5^ cells) were transfected with 10 µg of each plasmid DNA by calcium phosphate method. At 48 h, cells were washed with 1× PBS and lysed with 1% NP40 lysis buffer (100 mM Tris [pH 7.5], 150 mM NaCl, 20 mM EDTA, 1% NP40, 0.5% Na deoxycholate) containing protease inhibitors (Roche Diagnostics), followed by centrifugation at 20,000×g at 4°C for 30 min to obtain cell lystates [44]. Aliquots of cell lysates were added to non-reducing buffer (2% SDS, 0.5 M Tris [pH 6.8], 20% glycerol, 0.001% bromophenol blue [final concentration]) and subjected to 12% polyacrylamide gel electrophoresis (PAGE) and Western blot analysis [44]. The first Ab included mixed human sera consisting of a pool of 9 sera from dengue patients with secondary infection and each mAb. Cell lysates were also collected from C6/36 cells infected with mock, DENV1 (Hawaii strain), DENV2 (NGC strain), DENV3 (H87 strain), DENV4 (H241 strain), JEV (RP-9 strain) or WNV (NY99 strain), and subjected to 12% PAGE and Western blot analysis with each mAb to determine the binding specificity. Verification of similar amounts of antigen loading was done by Western blot analysis using mixed human sera, as described previously [44].

### Dot Blot Assay

Aliquots of cell lysates (in 1% NP40 lysis buffer) derived from the above transfectants were diluted in bromophenol blue containing 1× PBS and dot blotted by using a 96-dot formatted dot-blotter (Labrepco) to nitrocellulose membrane (Hybond-C; Amersham Biosciences). After blocking of the membrane with 4% milk in wash buffer, incubation with the first Ab (mixed mAbs consisting a pool of mAbs recognizing different epitopes, each mAbs or serum) and secondary Ab (horseradish peroxidase-conjugated anti-human or anti-mouse IgG) (Pierce), and final washing, the signals were detected by enhanced chemiluminescence reagents (Perkin Elmer life sciences) [44]. The intensities of E protein dots (dot blot assay) or bands (Western blot analysis) of wild type (WT) pCB-D1 and mutants were analyzed by imageQant (GE Healthcare, UK) [44]. The recognition index (R.I.) of a mAb to a mutant E protein was calculated as previously described [44], [48]. Briefly, R.I. = [intensity of mutant E dot (or band)/intensity of WT E dot (or band)] (recognized by a mAb)/[intensity of mutant E dot (or band)/intensity of WT E dot (or band)] (recognized by mixed mAbs or mixed sera).

### VLP-Capture ELISA

293T cells (1×10^5^ cells) were transfected with 10 µg of plasmid DNA as described above. At 48 h, culture supernatants were collected, clarified by centrifugation at 1,250×g for 20 min, filtered through a 0.22 µm pore-sized membrane (Sartorius), layered over a 20% sucrose buffer, and ultracentrifuged at 65,000×g at 4°C for 5 h to obtain pellets containing VLPs, which were resuspended in 30 µl TNE buffer [45]. Flat-bottom 96 well plate was coated with rabbit anti-sera against DENV1 at 4°C overnight, followed by blocking with 1% BSA in 1× PBS for 1 h. VLPs and mutant VLPs (at about 0.1 µg/ml, as determined by SDS-12% PAGE and Coomassie blue staining with BSA of known concentration as standards), serially two-fold dilutions of purified anti-E mAb, and anti-mouse IgG conjugated with HRP were added each at 37°C for 1 h, followed by TMB substrate and stop solution [27], [28]. Comparable amounts of WT and mutant VLPs added were confirmed by mixed mAbs, and the absorbance at wavelength of 450 nm (OD450) with reference wavelength of 650 nm was read. Binding curves, maximum binding (Bmax) and relative binding (% of Bmax) were determined by a nonlinear regression analysis with GraphPad Prism5 (GraphPad software Inc., CA).

### PRNT

Each anti-E mAb was purified by Protein G HP Spin Trap kit (GE Healthcare). Serially two-fold dilutions of purified anti-E mAb were mixed with 50 plaque forming unit (pfu) of DENV1 (Hawaii strain) at 37°C for 1 h, followed by inoculation to BHK-21 cells in 24-well plate (in duplicates) at 37°C for 2 h, overlay with MEM containing 2% FBS, antibiotic and 1% carboxymethyl cellulose) and incubation at 37°C for 5 to 7 days [38]. Cells were stained with 0.5% crystal violet; the plaques were counted and PRNT_50_ was presented as the lowest concentration that inhibited ≥50% of plaques.

### Epitope Analysis by Structure-Based Modeling

The program FirstGlance in Jmol version 1.44 (http://molvis.sdsc. edu/fgij/index.htm) was used to determine the locations of candidate epitope residues and distances between them (at the same or adjacent monomer) on E-E dimers. Similar analysis was also carried out by using the program UCSF chimera (http://www.cgl.ucsf.edu/chimera/) for E protein on the virus particles.

## Results

### Evaluation of the Binding Specificity of Multiple Anti-E mAbs to Flaviviruses

**Figure 1:**
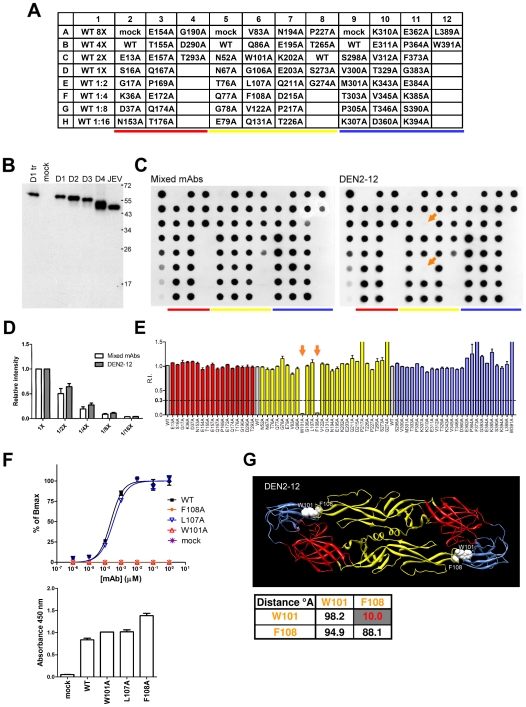
Specificity and epitope of flavivirus GR mAb DEN2-12. (A) Layout of the dot blot assay. (B) Binding specificity was examined by Western blot analysis as described in Methods. Lysates of 293T cells transfected with pCB-D1 (D1 tr) were also included. (C) Dot blot assay using lysates from 293T cells transfected with the WT pCB-D1 or each of the 67 alanine E mutants. Each membrane was probed with mAb DEN2-12 or mixed mAbs (a pool of mAbs recognizing different epitopes). The dots containing mutations in domains I, II and III are underlined by red, yellow and blue lines, respectively; the ID of each dot is shown in panel A. Two-fold dilutions of the WT lysates were dotted on row 1 to assess the exposure of each membrane. Arrows indicate mutants of epitope residues, which showed severe reduction (R.I.≤0.3) in binding by dot blot assay. One representative experiment of two was shown. (D) The relative intensities of WT dots in row 1 showed a linear decrease from 1× to 1∶16 dilution for membranes probed with mixed mAbs (open bar) and mAb DEN2-12 (closed bar). (E) The intensities of each dot were quantified to determine the R.I. as described in Methods. Data are means and standard errors of two experiments. (F) Capture ELISA was performed by using WT or mutant VLPs [28], which had severe reduction in binding by dot blot and Western blot analyses. Data are means and standard errors of duplicates from one representative experiment of two. Lower graph shows the amounts of mutant VLPs added, which were not less than that of WT. (G) Structure-based analysis by UCSF chimera program to determine the locations of and distance (°A) between epitope residues from the same (shaded) or adjacent monomer.

First, the binding specificity of 12 mouse anti-E mAbs was investigated by Western blot analysis using cell lysates derived from C6/36 cells infected by each of the 4 serotypes of DENV and JEV. MAbs DEN2-12, FL0232, FL0231, 4G2 and DEN4-4 recognized the E proteins of DENV1 to DENV4 and JEV, suggesting that they were flavivirus GR mAbs (Figure 1B and Figure S1). MAbs DEN2-9 and DEN3-3 recognized the E proteins of 4 DENV serotypes but not that of JEV, suggesting that they were CR mAbs, whereas mAbs DEN3-4 and DEN1-2 recognized the E proteins of DENV1 to DENV3, suggesting that they were sCR mAbs (Figure S1). MAbs DA6-7, DEN1-3 and FL0251 recognized DENV1 E protein only, suggesting that they were DENV1 TS mAbs.

### Epitope Mapping of Flavivirus GR and CR mAbs

To identify the epitopes recognized by these anti-E mAbs, site-directed mutagenesis was performed on a DENV1 prM/E expression construct, pCB-D1, to replace each of the 67 E residues, which were predicted to be surface-exposed or previously reported as epitopes of several mAbs, with an alanine [45], [48]. Since these mAbs recognized E protein only without significant background (Figure S1), we developed a dot blot assay for epitope mapping by blotting cell lysates derived from transfectants of each mutant in a 96-dot format, hybridized with each mAb or a pool of mixed mAbs, and examined the residues with loss of binding. To exclude the possibility of over-exposure, we dotted each membrane with two-fold serial dilutions of WT lysates; each membrane showed a linear decrease in intensity (Figure 1D). The intensity of each mutant dot recognized by mixed mAbs was generally comparable to that of the WT dot, suggesting that comparable amounts of E protein were dotted in the membrane (Figure 1C). Compared with that of the WT, the E binding activity of DEN2-12 was greatly reduced by two alanine mutants at the fusion loop of domain II (W101A and F108A) but not by other mutants, suggesting that these two residues were possibly the epitope of DEN2-12. We also calculated the R.I., which was the ratio of the intensity of the mutant E dot to that of WT dot recognized by a mAb divided by such ratio recognized by mixed mAbs [44], [48], and found a great reduction in R.I. for mutants W101A and F108A (Figure 1E). The results were further verified by Western blot analysis (Figure S2A). To further examine the binding of DEN2-12 to mutants W101A and F108A in the context of particles, we employed a previously described capture ELISA using WT and mutant VLPs [27], [28]. While DEN2-12 bound to mutant VLPs of L107A comparably to its binding to WT VLPs, it bound poorly to mutant VLPs of W101A or F108A (Figure 1F). Analysis of the locations of these residues by a structure-based modeling program revealed that the distance between these two residues from the same monomers was less than 30°A (Figure 1G), suggesting that residues W101 and F108 very likely constitute the epitope of DEN2-12.

**Figure 2:**
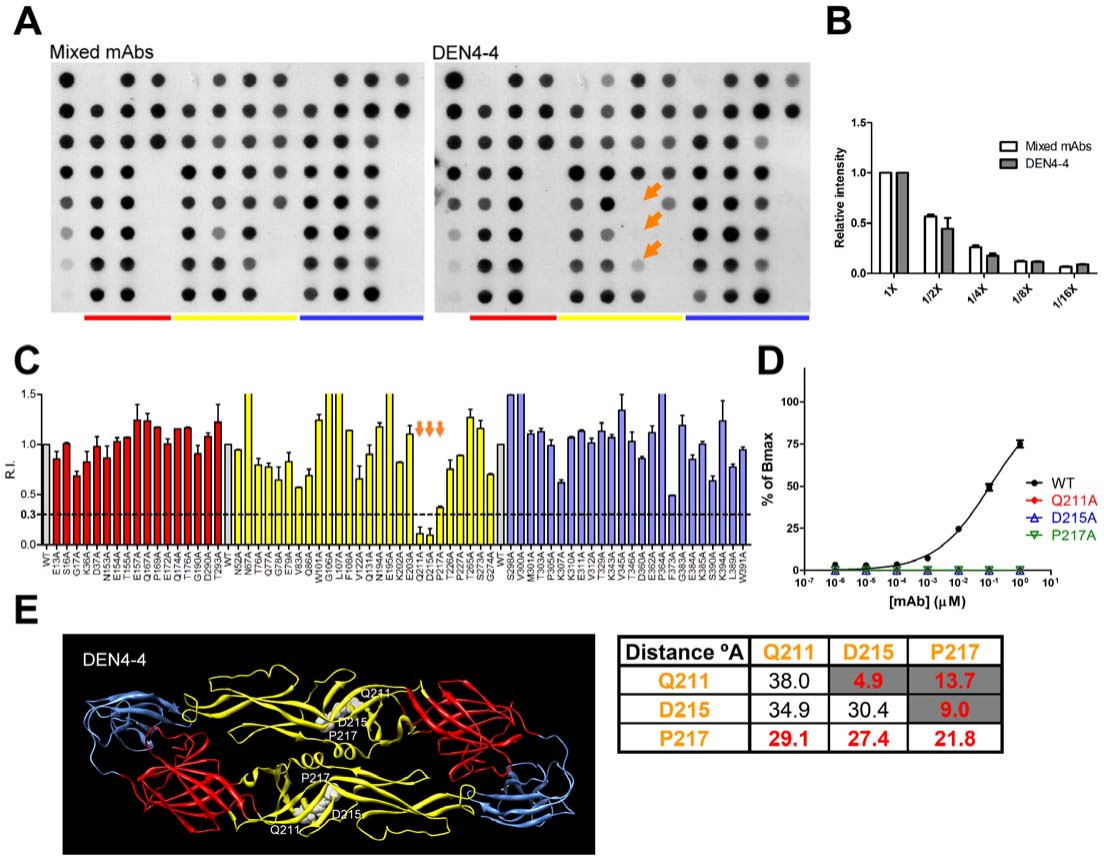
Epitope mapping of GR mAb DEN4-4. The results of (A, B, C) dot blot assay, (D) VLP-capture ELISA, and (E) structure based analysis of the locations of and distance (°A) between epitope residues from the same or adjacent monomer are presented as in Figure 1.

By using the same approach, the epitopes of the other three GR mAbs (FL0232, FL0231 and 4G2) were found to involve four fusion loop residues (W101A, G106A, L107A and F108A) with different combinations (Table 1). For another GR mAbs (DEN4-4), dot blot assay revealed that the E-binding activity was greatly reduced by three mutants (Q211A, D215A and P217A) at the central interface of domain II but not by fusion loop mutants (Figures 2A to 2C); this was further confirmed by Western blot analysis (Figure S2B) and VLP-capture ELISA (Figure 2D). Structure-based analysis revealed that the distances between these residues from the same or adjacent monomers were less than 30°A, suggesting that these three residues likely form an interdomain epitope at the domain II central interface (Figure 2E). This is a novel epitope for GR mAbs, since all the GR mAbs reported thus far were mainly mapped to the fusion loop [27], [28]. Interestingly, two CR mAbs were also found to have an interdomain epitope at the domain II central interface, including residues P217 and T265 for DEN2-9 and residue P217 for DEN3-3 (Table 1).

**Table 1:** Summary of the specificity, binding to alanine E mutants and PRNT_50_ of 12 mAbs.

mAbs	Class* (Specificity)	Binding**	Domain II†									
			Lateral ridge		fusion loop				central interface			
			V83A	Q86A	W101A	G106A	L107A	F108A	Q211A	D215A	P217A	T265A
DEN2-12	GR (DENV1,2,3,4,JEV)	dot blot WB			++ ++			++ ++				
FL0232	GR (DENV1,2,3,4,JEV)	dot blot WB			++ ++			++ ++				
FL0231	GR (DENV1,2,3,4,JEV)	dot blot WB			++ ++		++ ++	++ ++				
4G2	GR (DENV1,2,3,4,JEV)	dot blot WB			++ ++	++ ++	++ ++	++ ++				
DEN4-4	GR (DENV1,2,3,4,JEV)	dot blot WB							++ ++	++ ++	+ ++	
DEN2-9	CR (DENV1,2,3,4)	dot blot WB									++ ++	++ ++
DEN3-3	CR (DENV1,2,3,4)	dot blot WB									+ +	
DEN3-4	sCR (DENV1,2,3)	dot blot WB	+ +	+ +								
DEN1-2	sCR (DENV1,2,3)	dot blot WB										
FL0251	TS (DENV1)	dot blot WB	+ +									
DEN1-3	TS (DENV1)	dot blot WB	+ +									
DA6-7	TS (DENV1)	dot blot WB										

Domain III†												PRNT_50_ ‡
N-terminus			A strand				BC loop	FG loop		G strand		(µg/ml)
V300A	M301A	T303A	K307A	K310A	E311A	V312A	T329A	G383A	K385A	L389A	W391A	
												<6.25
												<12.5
												>100
												<25
												<50
												<50
												<12.5
					+ ++					++ ++	++ ++	<1.56
++ ++	++ ++	++ ++					++ ++	++ +				<1.25
			++ ++	++ ++						++ +		<6.25
++ ++	++ ++	++ ++					++ ++	++ ++	+ +			<0.78
			++ ++	++ ++		+ +				++ ++	++ ++	<1.25

*Classes of mAbs include GR (group-reactive), CR (complex-reactive), sCR (subcomplex-reactive) and TS (type-specific). Specificity was determined by Western blot (WB) analysis using lysates derived form DENV1, 2, 3, 4 or JEV-infected C6/36 cells as described in Methods.

**Alanine E mutants with consistent reduction in binding by dot blot and WB analyses are shown. ++: severe reduction in binding (reduction in R.I.≥70%);

+: moderate reduction in binding (50%≤reduction in R.I.<70%); underlined: reduced binding tested by VLP-capture ELISA.

**†:** Different regions (strand, loop, etc.) of domains II and III are defined based on the X-ray structure of DENV E protein [7], [24], [33].

**‡:** PRNT_50_ was presented as the lowest concentration that inhibited ≥50% of plaques [38].

### Epitope Mapping of sCR and TS mAbs

For the TS mAb FL0251, four epitope residues were identified, including severe reduction in binding (R.I.≤0.3) of 3 mutants at the A strand (K307A, K310A) and G strand (L389A) of domain III and moderate reduction in binding (0.3<R.I.≤0.5) of a mutant at the lateral ridge (V83A) of domain II (Figures S3A to S3D). Mutants F373A and W391A, which showed severe and moderate reduction in binding by dot blot assay, respectively, were excluded as epitope residues by VLP-capture ELISA (Figure S3D). This is probably related to different accessibility of these two residues in E protein solublized by NP40 lysis buffer and in the context of particles. Structure-based analysis revealed that the distances between the three domain III residues from the same monomer as well as those between domain III residue (K310) and domain II residue (V83) from the adjacent monomer were less than 30°A, suggesting an interdomain epitope (Figure S3E).

Similarly, the epitopes of another TS mAb (DEN1-3) and a sCR mAb (DEN3-4) were interdomain epitopes involving residues at domain III and domain II lateral ridge (Table 1). The epitopes of another TS mAb (DA6-7) and a sCR mAb (DEN1-2) were found to involve domain III residues only (Table 1).

### Relationship between Neutralizing Potency and Epitope

We next examined the neutralizing potency of these 12 mAbs. In agreement with previous reports, the neutralizing potency of TS or sCR mAbs was generally greater than that of CR or GR mAbs (Table 1) [29], [30], [32]. However, the neutralizing potency of our TS or sCR mAbs (PRNT_50_ in the range of µg/ml) was much lower than that of the potent TS or sCR mAbs reported recently (PRNT_50_ in the range of ng/ml) [33]–[35]. It is worth noting that these potent neutralizing mAbs were generated by a novel immunization protocol involving two DENV challenges in IFN-α/β R -/- C57BL/6 mice and a booster with recombinant domain III, whereas previous protocols (including ours) involved immunization of WT BALB/c mice with DENV [29], [30], [32], [38]–[42]. To investigate if these potent neutralizing mAbs recognize epitopes different from those recognized by less potent mAbs, we compared the epitopes and PRNT_50_ of mAbs generated from two different protocols using DENV1 or DENV2 as immunogen (Table S1). For the mAbs against DENV2, both groups recognized residues in the A strand, BC loop and G strand of domain III (Table 2). Interestingly, the potent neutralizing mAbs (PRNT_50_≤311 ng/ml) recognized either four or more residues in the A strand, residues in the C strand/CC′ loop, or multiple residues in both A strand and BC loop [34], whereas less potent mAbs commonly recognized residues in the FG loop [29], [32]. Since the epitopes of most of these DENV2 mAbs were identified by the same assay, it is unlikely that the epitopes identified were biased by the methods utilized [32], [34]. For the mAbs against DENV1, the potent neutralizing mAbs (PRNT_50_≤590 ng/ml) recognized four residues in the A strand, three or more than three residues in BC loop, residues in C strand/CC′ loop, DE loop, EF loop/F strand, or G strand only [33] (Table 2).

**Table 2:** Comparison of epitopes and neutralization potency of mAbs recognizing domain III of DENV E protein.

	Potent neutralizing mAbs*	Less potent neutralizing mAbs*
DENV2 as immunogen		
Common features	residues in A strand, BC loop and G strand	residues in A strand, BC loop and G strand
Unique features	≥4 residues in A strand (such as residues 303, 305, 307, 309)	residues in FG loop
	residues in C strand/CC′ loop (such as residues 336, 340, 346)	
	multiple residues in both A strand and BC loop	
DENV1 as immunogen		
Common features	residues in N-terminus, A strand, BC loop and G strand	residues in N-terminus, A strand, BC loop and G strand
Unique features	4 residues in A strand (such as residues 303, 305, 307, 309)	
	residues in C strand/CC′ loop (such as residues 334, 343)	
	≥3 residues in BC loop (such as residues 328, 329, 330, 332)	
	residues in DE loop (such as residues 361, 362, 364), EF loop/F strand (such as residues 370, 375), or G strand only (such as residues 390, 391)	

*Potent neutralizing mAbs against DENV2 and DENV1 were defined by PRNT_50_ ≤311 µg/ml and ≤590 µg/ml, respectively. Most of the potent neutralizing mAbs were generated by immunization of IFN-α/β R -/- C57BL/6 mice with DENV twice and booster with recombinant domain III [33], [34] , whereas most of the less potent neutralizing mAbs were generated by immunization of WT BALB/c mice [29], [30], [32], [38]–[42].

### Predominant Epitopes in Polyclonal Human Sera

**Figure 3:**
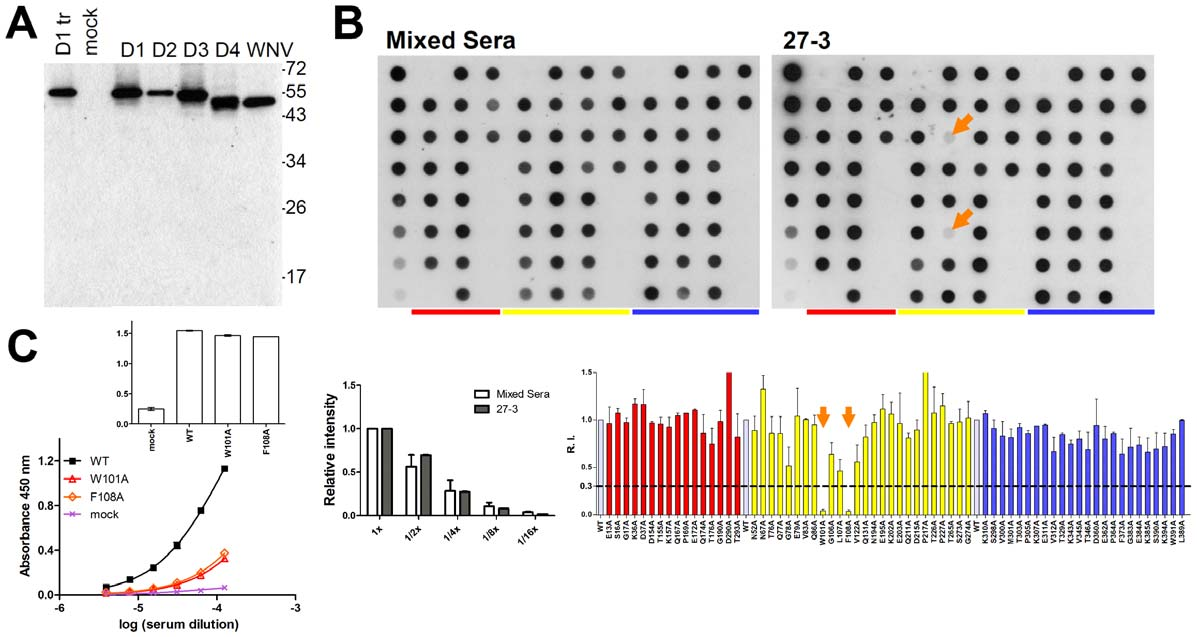
Binding specificity and predominant epitope recognized by anti-E Abs in serum from a DENV1 case. (A) Binding specificity was examined by Western blot analysis as described in Methods. Lysates of 293T cells transfected with pCB-D1 (D1 tr) were also included. (B) Dot blot assay presented as in Figure 1A and 1C to 1E (except that WT dot in row 8C and 153NA dot in row 2H were omitted) was probed with the tested serum or mixed sera, which consisted of a pool of 9 sera from confirmed dengue patients [44]. The relative intensities of two-fold dilutions of WT dots in row 1 were presented as in Figure 1D. R.I. of each mutant was shown as in Figure 1E. One representative experiment of two was shown. (C) Capture ELISA using WT or mutant VLPs was presented as in Figure 1F. Upper graph in panel C shows comparable amounts of WT and mutant VLPs added.

To further explore the possibility that this assay can be employed to investigate the predominant epitope of anti-E Abs in polyclonal human sera, we examined serum from a confirmed DENV1 secondary infection case. As shown in Figure 3A, Western blot analysis revealed that this serum can recognize not only the E protein of DENV1 but also that of DENV2, 3, 4 and WNV. Since this serum recognized E protein without significant background, we used the same serum dilution in the dot blot assay. Compared with that of the WT, the E binding activity was greatly reduced by two alanine mutants at the fusion loop (W101A and F108A) but not by other mutants, suggesting that these two residues possibly constitute a predominant epiotpe in this polyclonal serum (Figure 3B). This was further verified by VLP-capture ELISA (Figure 3C). Analysis of the sera from other two cases of secondary infection by DENV2 (Figures S4A to S4C) or DENV3 (Figures S4D to S4F) revealed that the E binding activity was severely reduced by mutants W101A and F108A in both dot blot assay and VLP-capture ELISA, suggesting that fusion loop residues form a predominant epitope in these two polyclonal sera. For the DENV2 case, moderate reduction in binding was also found in mutants T293A at domain I and V122A at domain II. Table 3 summarizes the results of 14 secondary infection cases including six DENV1 cases, three DENV2 cases and five DENV3 cases. The E binding activities were greatly or moderately reduced by mutations in the fusion loop including W101A and F108A and/or L107A and G106A, suggesting these are the predominant epitopes. Interestingly, the E binding activities were also moderately affected by some mutations outside of the fusion loop such as G78A, V122A, D290A and T293A. The distance between fusion loop residues and residue G78 of the same monomer was less than 30°A and that between fusion loop residues and D290 or T293 from the adjacent monomer was close to 30°A, suggesting that they likely form a predominant epitope.

**Table 3:** Summary of predominant epitopes recognized by anti-E Abs in human sera after DENV infection.

Patient ID	Serotype and immune status*	Disease**	Sampling time (after onset)	Predominant epitopes† recognized by anti-E Abs
854	Secondary DENV1	DF	19 d	W101, F108
895	Secondary DENV1	DF	35 d	W101, F108
436	Secondary DENV1	DF	18 d	W101, F108
774	Secondary DENV1	DF	45 d	W101, (L107)
923	Secondary DENV1	DF	34 d	W101, F108 (L107)
27-3	Secondary DENV1	DF	3 months	W101, F108 (L107)
45	Secondary DENV2	DF	6 years	W101, F108, (V122, T293)
80	Secondary DENV2	DF	6 years	(F108)
97	Secondary DENV2	DF	6 years	(W101)
92	Secondary DENV3	DF	2 years	(W101, F108)
102	Secondary DENV3	DF	6 years	W101, F108
70	Secondary DENV3	DF	1.5 years	W101, F108
87	Secondary DENV3	DF	2 years	F108, (W101, G78, D290)
95	Secondary DENV3	DF	1.5 years	W101, F108, (L107, V122)

*Primary or secondary infection was determined by PRNT_50_ as described in Methods.

**DF, dengue fever according to WHO case definition [2].

**†:** Predominant epitopes recognized by anti-E Abs in polyclonal human sera were identified by dot blot assay and residues with severe (reduction in R.I.≥70%) or moderate (50%≤reduction in R.I.<70%, shown in parenthesis) impairment in binding were shown.

## Discussion

In this study we developed a high throughput dot blot assay to investigate the epitopes on E protein recognized by mouse mAbs and polyclonal human sera; the epitope residues were further verified by a VLP-capture ELISA assay to ensure accurate epitope mapping. Of the 12 mAbs studied, three mAbs recognized a novel epitope involving residues at the domain II central interface (residues Q211, D215D, P217) and three mAbs recognized residues at both domain III and the lateral ridge of domain II, suggesting a more frequent presence of interdomain epitopes than previously appreciated. Identification of such epitopes on the virion surface typically requires a cryo-EM study of virions in the presence of Fab of mAb, as recently demonstrated by the study of a human mAb (CR4354) spanning domains III and I of adjacent E monomers on WNV virions [49]. Compared with the epitopes of mAbs generated by traditional protocols, the epitopes of potent neutralizing mAbs generated by a new protocol involving IFN-α/β R -/- C57BL/6 mice showed several interesting and unique features (Table 2). Together, these findings have implications for future development of epitope-specific diagnostics and epitope-based dengue vaccine.

Previous studies of epitope mapping were primarily based on either yeast surface-display library containing domain III or domain I/II alone or bacterially expressed recombinant domain III; the possibility of interdomain epitopes on DENV E protein cannot be identified by either system [29], [30], [32]–[35]. In the bacterial expression system, fusion proteins containing maltose binding protein and WT or mutant domain III were coated on ELISA plate under alkaline buffer (pH 9.0) [29]–[30], raising the concern whether the conformation of domain III was preserved. The yeast surface-display system involving screening for mutant library by flow cytometry required a time-consuming process of sorting, sequencing and confirmation [32]–[35]. In a newly adopted dot blot assay, we prepared 67 alanine-substitution mutants in 1% NP40 lysis buffer without SDS or boiling, a condition similar to RIPA lysis buffer containing non-ionic detergent to preserve the conformation of membrane protein, and dotted in the 96-dot format. A linear decrease in the intensity of two-fold serial dilutions of WT lysate dots on each membrane suggested that the assay signal is sensitive to the serial decrease in the amount of E protein and excluded the possibility of over-exposure. Moreover, the epitope residues identified were verified by VLP-capture ELISA to assess the binding in the context of particles; both assays were required for accurate epitope mapping. It is worth noting that since some partially occluded epitopes were shown to be exposed after conformational movement of E protein [50], certain interesting and potentially important epitope residues might not be represented by this panel of 67 residues. Nonetheless, our dot blot assay is a convenient and high throughput method to investigate both intra and interdomain epitopes on DENV E protein and has tremendous applications. Using this approach to study mAbs, our findings that interdomain epitopes were more frequent than previously appreciated suggest that subunit vaccines containing recombinant domain III or domain I/II alone are probably not optimal to induce neutralizing Abs compared with immunogen containing entire E protein. Consistent with this interpretation, a recent study of different subunit vaccine candidates for WNV revealed that domain III alone elicited at least 15-fold lower neutralizing activity compared with inactivated virion [51].

Recent studies of polyclonal human sera and mAbs derived from dengue cases revealed that anti-E Abs were highly cross-reactive [28], [44], [52]–[54]. Studies of human sera by the approach of loss-of-binding to variant E protein containing mutations in selected residues in the domain II or domain III reported that a significant proportion of anti-E Abs after DENV infection recognized the fusion loop of domain II, whereas only a minor proportion recognized domain III [28], [44]. A similar trend was also observed in the studies of human sera [55] and mAbs repertoire after WNV infection [56]. However, a systematic approach was lacking to investigate the predominant epitopes recognized by anti-E Abs in polyclonal sera. Using our approach to examine 14 dengue sera from secondary infection cases, we demonstrated for the first time that the predominant epitopes on E protein recognized by polyclonal sera can be quickly identified. Our findings suggested that fusion loop residues (such as W101, F108, L107 and G106) together with some non-fusion loop residues (such as G78, D290 or T293) in the same or adjacent monomer likely constitute a predominant epitope in these cases. It is worth noting that the symmetry of VLPs is not identical to that of virions. Several studies using conformation-sensitive neutralizing mAbs against TBEV E protein have shown that the structure of E protein on VLPs was very similar to that on virions and that E proteins on both VLPs and virions displayed closely related changes in conformational and oligomeric state when exposed to acidic pH [14]–[16]. Since these mAbs do not recognize multi-protein spanning or interdimer epitopes on the surface of virions, the possibility that some anti-E Abs recognizing such epitopes on virions might be missed by VLP-capture ELISA can not be completely ruled out, especially when measuring complex samples such as sera.

Cross-reactivity among different flaviviruses has been an obstacle for serological diagnosis to distinguish various flaviviral infections [4], [17], [20]. Studies introducing mutations to the highly conserved fusion loop residues (such as G106 and L107) have led to the development of type-specific serodiagnostic assays for DENV, JEV and SLEV [17], [20]. A better understanding of the epitopes that contribute to flaviviral cross-reactivity would facilitate the improvement in flaviviral serodiagnosis and epitope-specific diagnostics. Our analysis suggests that in addition to fusion loop residues, residues at the domain II central interface (Q211, D215 and P217), an epitope for GR and some CR mAbs, may be considered for further reduction of cross-reactivity.

Recent studies of high-resolution structures of flaviviral virions in the presence or absence of Fab of mAb and the mechanisms involved in neutralization suggested that flavivirus neutralization is a multiple-hit phenomenon, in which neutralization occurs only when the binding of Abs to virions exceeds a stoichiometric threshold [57]–[60]. The potency of a neutralizing Ab is determined by several factors including the affinity of Abs and accessibility of the epitopes, which were affected by the extent of virion maturation and conformational change of E protein on the virion [58], [59]. Elucidation of the epitopes recognized by potent anti-DENV neutralizing mAbs have implication for future strategy of epitope-based subunit vaccine against DENV.

**Figure 4:**
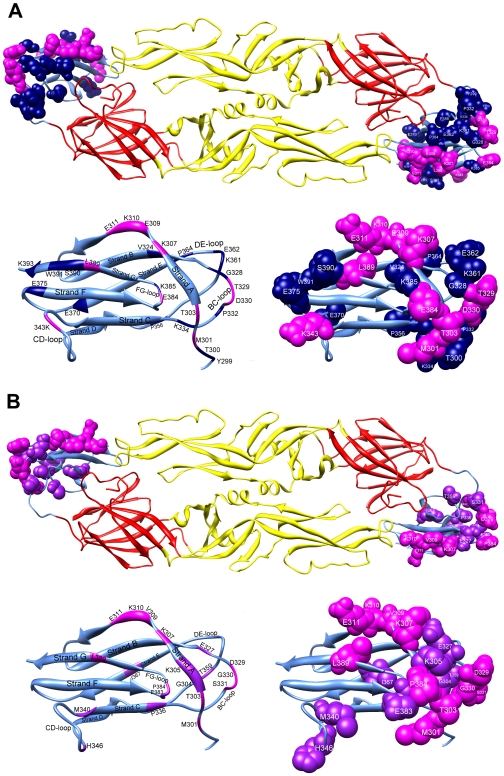
Location of epitope residues on E protein recognized by potent neutralizing mAbs. Epitope residues of mAbs against DENV1 (A) and DENV2 (B). Epitope residues are highlighted with dark blue (DENV1 residues), purple (DENV2 residues), or magenta (DENV1 and DENV2 residues at the same position). Top view of E-E dimers (upper) and side view of domain III (lower right) with ribbon presentation of ß-strands and loops (lower left) are shown by the program UCSF chimera.

Our analysis of the epitopes recognized by potent and less potent neutralizing mAbs revealed multiple overlapping residues in A strand and BC loop of domain III. However, several potent neutralizing mAbs recognized more than four residues in A strand (mAbs DV2-76, DV2-70, DV2-106, DV2-96, 1A1D-2, 1F1 and E99), BC loop (mAbs E101 and E103), or multiple residues in both A strand and BC loop (mAbs E90, 3H5 and 6B6-10), suggesting that increased affinity to these epitope residues may account for the increased potency [32]–[34] (Table S1). Other potent neutralizing mAbs recognized certain residues not found in less potent mAbs such as C strand/CC′ loop (mAbs DV2-77, DV2-73, DV2-104 and DV2-87), suggesting unique epitope residues of potent neutralizing mAbs [34] (Table S1). Notably, comparing the epitopes recognized by these potent neutralizing mAbs against DENV2 and DENV1 revealed both common and different features (Table 2), suggesting difference in the epitopic structure between DENV1 and DENV2. Figure 4 highlighted the locations of epitope residues recognized by these potent neutralizing mAbs. Detailed analysis of the extent of conservation of these epitope residues might provide important information for new strategies to elicit highly TS or CR potent neutralizing Abs using different immunogens. Moreover, future studies to delineate the epitopes recognized by human neutralizing mAbs and neutralizing Abs in polyclonal human sera would provide another piece of critical information for rationale design of subunit vaccine against DENV and add new insight into our understanding of protective humoral immune responses against DENV during the natural course of infection.

## Supporting Information

Figure S1 **Binding specificity of 12 mouse anti-E mAbs.** Western blot analysis was performed by using cell lysates derived from C6/36 cells infected with each of the 4 DENV serotypes or JEV. Lysates of 293T cells transfected with pCB-D1 (D1 tr) were also included.
(TIF)

Figure S2 **Western blot analysis.** Cell lysates derived from 293T cells transfected with WT pCB-D1 or each of the 67 alanine-substitution E mutants were probed with mAbs (A) DEN2-12, (B) DEN4-4, (C) FL0251 and mixed sera, which consisted of a pool of 9 sera from confirmed dengue patients. R.I. of each mutant was determined as described in Methods [44], [48].
(TIF)

Figure S3 **Epitope mapping of TS mAb FL0251.** The results of (A, B, C) dot blot assay, (D) VLP-capture ELISA, and (F) structure based analysis of the location of and distance (°A) between epitope residues from the same or adjacent monomer are presented as in Fig. 2. Arrow heads indicate mutants of epitope residues, which showed moderate reduction in binding (0.3<R.I.≤0.5) by dot blot and Western blot analyses.
(TIF)

Figure S4 **Binding specificity and predominant epitope recognized by anti-E Abs in human sera from dengue cases.** Shown are cases of DENV2 (A,B,C), and DENV3 (D,E,F). (A,D) Binding specificity was examined by Western blot analysis as described in Methods. Lysates of 293T cells transfected with pCB-D1 (D1 tr) were also included. (B,E) Dot blot assay presented as in Fig. 1A and 1C to 1E (except that WT dot in row 8C and 153NA dot in row 2H were omitted) was probed with the tested serum or mixed sera, which consisted of a pool of 9 sera from confirmed dengue patients [44]. The relative intensities of two-fold dilutions of WT dots in row 1 were presented as in Fig. 1D. R.I. of each mutant was shown as in Fig. 1E. One representative experiment of two was shown. (C,F) Capture ELISA using WT or mutant VLPs was presented as in Fig. 1F. Upper graph in panel C shows comparable amounts of WT and mutant VLPs added.
(TIF)

Table S1 **Comparison of epitopes, neutralization potency and immunization protocol of CR/sCR and TS mAbs recognizing domain III of DENV E protein.**
(DOC)
