# Supplementary figures and images for: Analysis of Epitopes on Dengue Virus Envelope Protein Recognized by Monoclonal Antibodies and Polyclonal Human Sera by a High Throughput Assay
Source: PLoS Negl Trop Dis. 2012 Jan 3;6(1):e1447. doi: 10.1371/journal.pntd.0001447 (PMC3250511; doi:10.1371/journal.pntd.0001447)

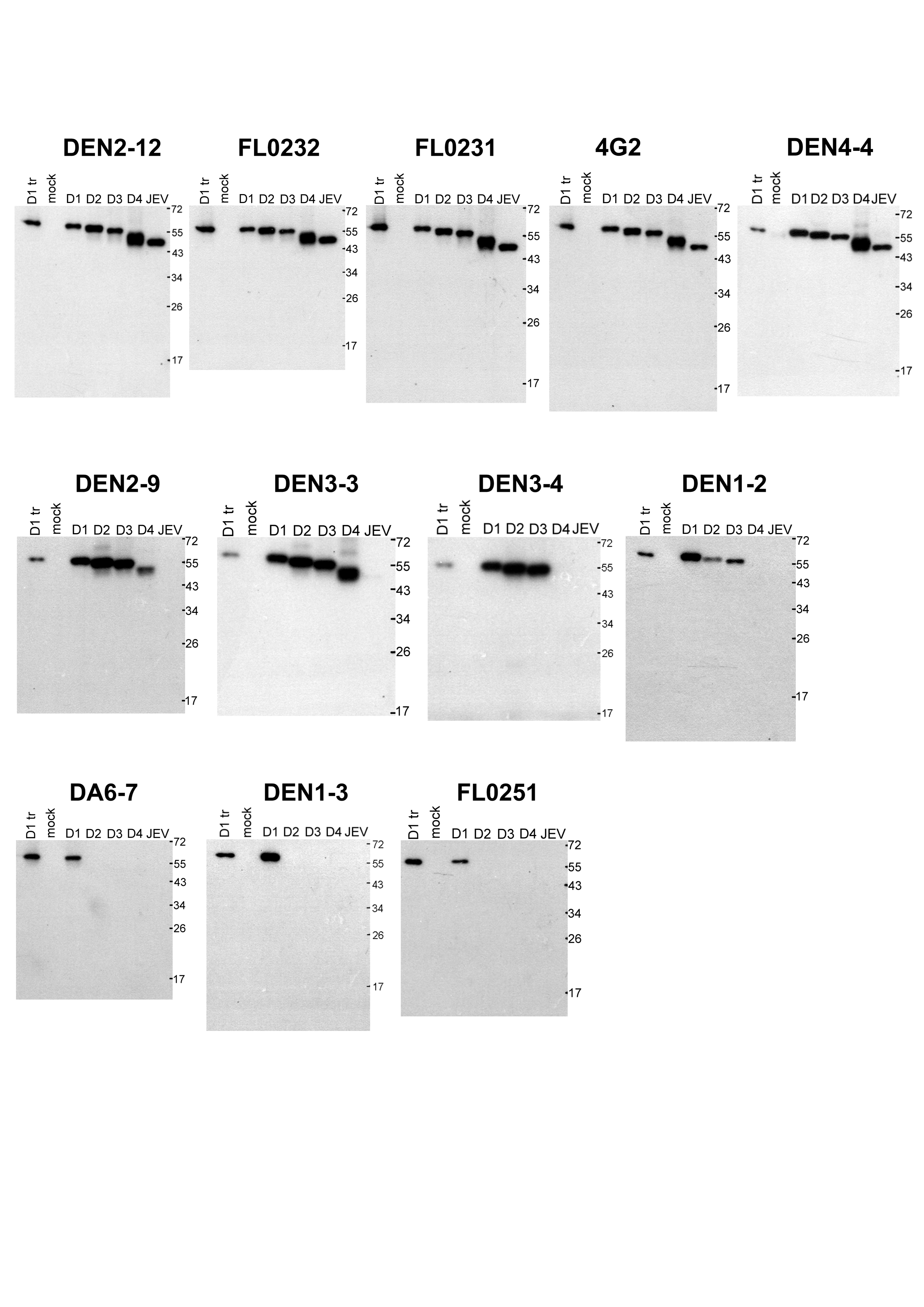

Supplement: Figure S1 — Binding specificity of 12 mouse anti-E mAbs. Western blot analysis was performed by using cell lysates derived from C6/36 cells infected with each of the 4 DENV serotypes or JEV. Lysates of 293T cells transfected with pCB-D1 (D1 tr) were also included. (TIF) [file pntd.0001447.s001.tif]

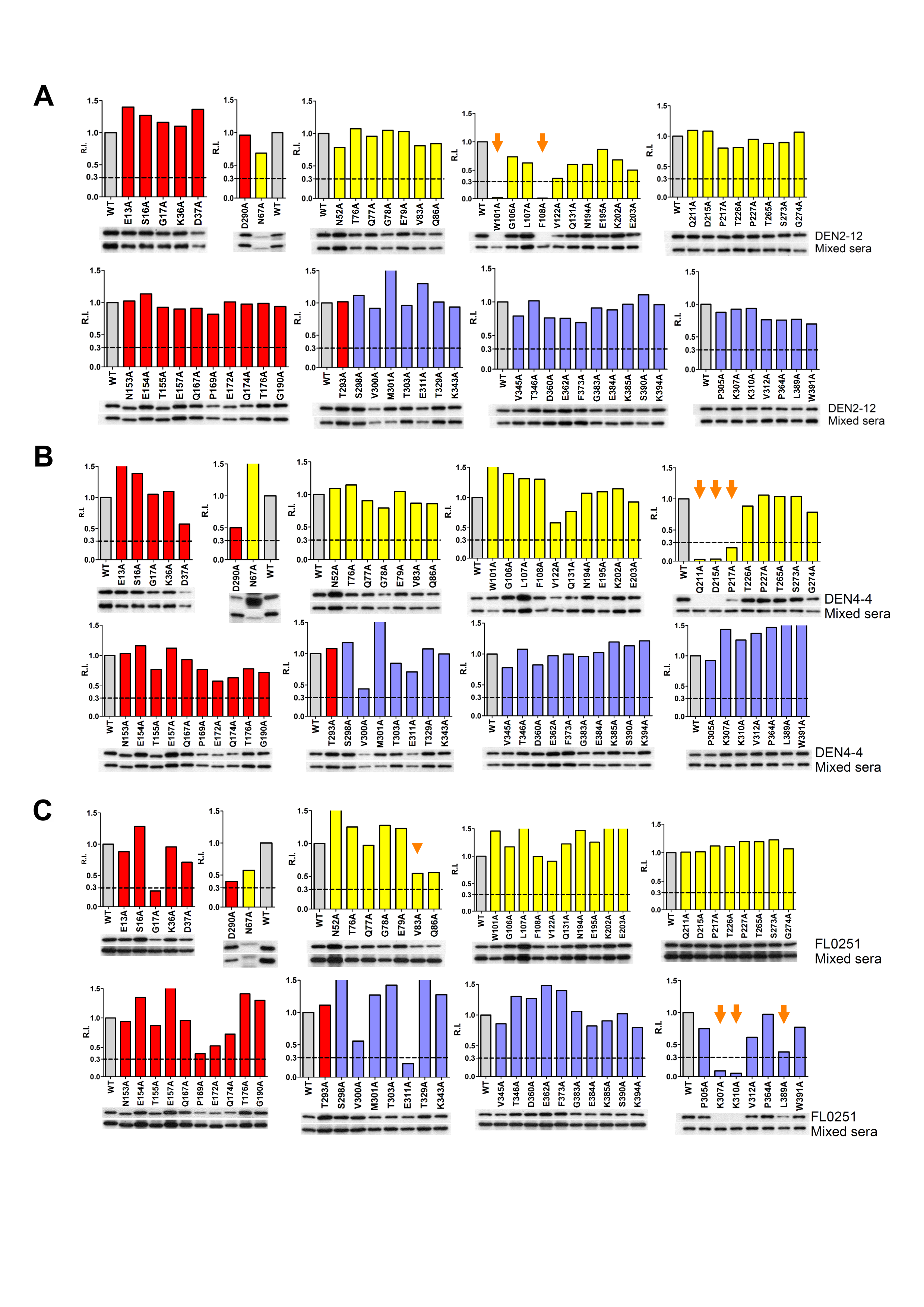

Supplement: Figure S2 — Western blot analysis. Cell lysates derived from 293T cells transfected with WT pCB-D1 or each of the 67 alanine-substitution E mutants were probed with mAbs (A) DEN2-12, (B) DEN4-4, (C) FL0251 and mixed sera, which consisted of a pool of 9 sera from confirmed dengue patients. R.I. of each mutant was determined as described in Methods [44], [48]. (TIF) [file pntd.0001447.s002.tif]

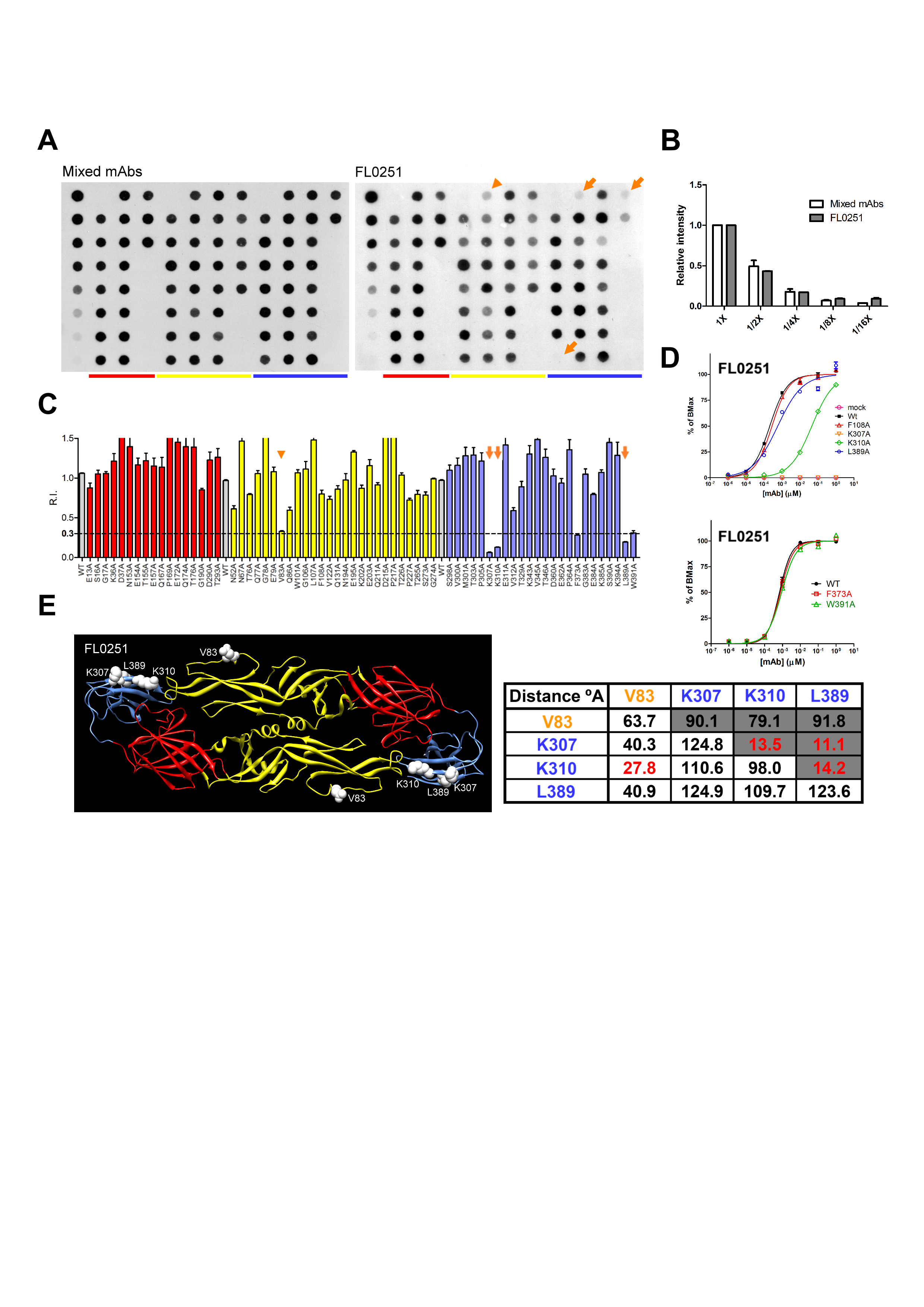

Supplement: Figure S3 — Epitope mapping of TS mAb FL0251. The results of (A, B, C) dot blot assay, (D) VLP-capture ELISA, and (F) structure based analysis of the location of and distance (°A) between epitope residues from the same or adjacent monomer are presented as in Fig. 2. Arrow heads indicate mutants of epitope residues, which showed moderate reduction in binding (0.3<R.I.≤0.5) by dot blot and Western blot analyses. (TIF) [file pntd.0001447.s003.tif]

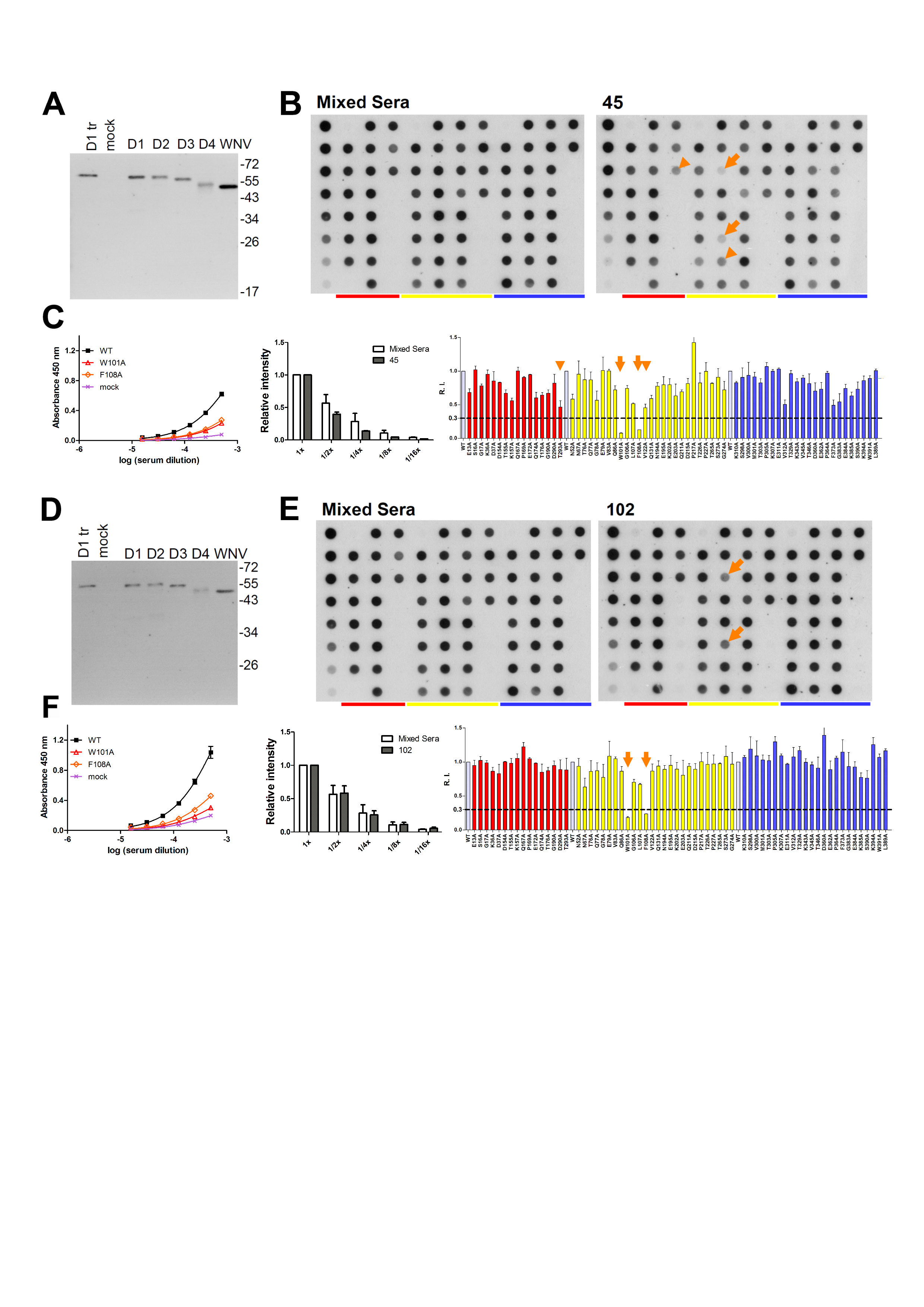

Supplement: Figure S4 — Binding specificity and predominant epitope recognized by anti-E Abs in human sera from dengue cases. Shown are cases of DENV2 (A,B,C), and DENV3 (D,E,F). (A,D) Binding specificity was examined by Western blot analysis as described in Methods. Lysates of 293T cells transfected with pCB-D1 (D1 tr) were also included. (B,E) Dot blot assay presented as in Fig. 1A and 1C to 1E (except that WT dot in row 8C and 153NA dot in row 2H were omitted) was probed with the tested serum or mixed sera, which consisted of a pool of 9 sera from confirmed dengue patients [44]. The relative intensities of two-fold dilutions of WT dots in row 1 were presented as in Fig. 1D. R.I. of each mutant was shown as in Fig. 1E. One representative experiment of two was shown. (C,F) Capture ELISA using WT or mutant VLPs was presented as in Fig. 1F. Upper graph in panel C shows comparable amounts of WT and mutant VLPs added. (TIF) [file pntd.0001447.s004.tif]
