# Supplementary material for: Analysis of Epitopes on Dengue Virus Envelope Protein Recognized by Monoclonal Antibodies and Polyclonal Human Sera by a High Throughput Assay
Source: PLoS Negl Trop Dis. 2012 Jan 3;6(1):e1447. doi: 10.1371/journal.pntd.0001447 (PMC3250511; doi:10.1371/journal.pntd.0001447)
Supplement: Table S1 — Comparison of epitopes, neutralization potency and immunization protocol of CR/sCR and TS mAbs recognizing domain III of DENV E protein. (DOC) [file pntd.0001447.s005.doc]

**Table S1.** Comparison of epitopes, neutralization potency and immunization protocol of

CR/sCR and TS mAbs recognizing domain III of DENV E protein.

| Immunization of BALB/c mice^*^ | | | | |  | Immunization of IFN-α/β R -/- C57BL/6 mice^**^ | | | | |
| --- | --- | --- | --- | --- | --- | --- | --- | --- | --- | --- |
| mAbs | Class^†^ | Epitope^‡^ | PRNT_50_^§^ | Ref. |  | mAbs | Class^†^ | Epitope^‡^ | PRNT_50_^§^ | Ref. |
| DENV1 as immunogen: | | | | |  | DENV1 as immunogen: | | | | |
| DEN1-2 | sCR | 300, 301, 303, 329, 383 | 1250 |  |  | E102 | CR | 299, 300, 301, 328, 330, 332, 334, (324) | 1.1 | [33] |
| DA6-7 | TS | 307, 310, 389, 391, (312) | 1250 | [39] |  | E90 | sCR | 300, 328, 332, (303, 307, 309) | 1.9 | [33] |
| DEN1-3 | TS | 300, 301, 303, 329, 383 (83, 385) | 780 |  |  | E98 | sCR | 375, 390, (301, 310) | 13 | [33] |
|  | | | | |  | E99 | sCR | 309, 310, 311, (307) | 11 | [33] |
| DENV2 as immunogen: | | | | |  |  |  |  |  |  |
| 5A2-7 | CR | 317, (304, 310, 315, 383) | >10^5^ | [32] |  | E106 | sCR | 310, 328, 329, 330, 361, 362, 364,  385, (332, 384) | 0.6 | [33] |
| 13D4-1 | CR | 317, (310, 359) | >10^5^ | [32] |  | E113 | sCR | 299, 300, 301, 310, 328, 332, 334, (356) | 1.3 | [33] |
| 9D12 | sCR | 304, 305, 307, 310, 384, (303, 329, 390) | 2000 | [32] |  | E100 | TS | 307, 329, 330, 361, 362, (300, 301, 310, 324, 332, 370, 393) | 24 | [33] |
| 1A1D-2 | sCR | 304, 305, 307, 310, (384) | 300 | [32] |  | E95 | TS | 390, 391 | 14 | [33] |
| 1F1 | TS | 303, 304, 330, 383, 384, (307, 310, 327, 329) | 10 | [32] |  | E101 | TS | 303, 328, 329, 330, 332 | 0.8 | [33] |
| 3H5-1 | TS  TS | 304, 383, 384, (307, 310, 327, 329)  305, 384, (329, 332) | 180 | [32]  [29] |  | E103 | TS | 303, 328, 329, 330, 332 | 0.7 | [33] |
| M8051122 | TS  TS | 304, 310, 383, 384, (307, 323)  305, 384 | 2000  530 | [32]  [29] |  | E104 | TS | 310, 311, 375, 385, 390, 391, 393, (328, 330, 361, 362) | 590 | [33] |
| 9F16 | TS  TS | 304, 383, 384, (310)  305, 384, (307, 327, 329) | 800 | [32]  [29] |  | E105 | TS | 329, 332, 361, 362, (307, 310, 324, 330, 343, 364, 390) | 0.5 | [33] |
| 2Q1899 | TS  TS | 304, 383, 384, (307, 310, 329, 390)  305, 384, (332) | 5000  790 | [32]  [29] |  | E108 | TS | 389, (311, 370) | 70 | [33] |
| 6B6-10 | TS | 304, 329, 330, 331, 384, (303, 310, 327) | 70 | [32] |  | E112 | TS | 390, 391 | 42 | [33] |
| 9A3D-8 | TS | 304, 307, 327 | 30 | [32] |  | DENV2 as immunogen: | | | | |
| 9F11 | TS | 304, 383, 384, (310, 327) | 2000 | [32] |  | DV2-77 | CR | 336, 340, 346, (301, 303) | 200 | [34] |
|  |  |  |  |  |  | DV2-76 | sCR | 305, 307, 309, (310, 327) | 29 | [34] |
|  |  |  |  |  |  | DV2-70 | TS | 305, 307, 309, 327, 389, (303, 330) | 168 | [34] |
|  |  |  |  |  |  | DV2-73 | TS | 336, 340, 346 | 92 | [34] |
|  |  |  |  |  |  | DV2-106 | TS | 305, 307, 327, 330, 389, (303, 309, 311, 359, 367) | 311 | [34] |
|  |  |  |  |  |  | DV2-96 | TS | 305, 307, 309, 389, (303, 327) | 67 | [34] |
|  |  |  |  |  |  | DV2-104 | TS | 336, 340, 346 | 76 | [34] |
|  |  |  |  |  |  | DV2-87 | TS | 336, 340, 346 | 76 | [34] |

^*^ BALB/c mice were immunized with DENV1 [38] or DENV2 [29,30,32,39-42], and subjected to generation of hybridoma and screening.

^**^ IFN-α/β R -/- C57BL/6 mice were infected with DENV1 (16007 strain) or DENV2 (16681 plus NGC strains), reinfected and boosted with

recombinant domain III, and subjected to generation of hybridoma and screening as described previously [33,34].

^†^ GR: flavivirus group-reactive; CR: complex-reactive; sCR: subcomplex-reactive; TS: type-specific.

^‡^ Epitope residues with severe (reduction ≥ 70% or 80% [32-34] or relative Kd ≥ 10 folds [17] and moderate (50% ≤ reduction < 70% or 80%

[32-34] or 4 folds ≤ relative Kd < 10 folds [29], shown in parenthesis) impairment in binding are shown. Light blue: N-terminus;

pink: A strand; light green: BC loop; red: C strand/CC’ loop; orange: DE loop; green: EF loop/F strand; brown: FG loop; blue: G strand.

^§^ PRNT_50_ of DENV1 or DENV2 of the homologous strain [33,34] or of the same genotype [29,32] was shown as ng/ml.
